# Evaluation of the nitrogen content during the new-shoot-growing stage in apple leaves using two-dimensional correlation spectroscopy

**DOI:** 10.1371/journal.pone.0186751

**Published:** 2017-10-26

**Authors:** Lulu Gao, Xicun Zhu, Cheng Li, Lizhen Cheng

**Affiliations:** 1 College of Resources and Environment, Shandong Agricultural University, Taian, Shandong, China; 2 Key Laboratory of Agricultural Ecology and Environment, Shandong Agricultural University, Taian, Shandong, China; United States Department of Agriculture, UNITED STATES

## Abstract

The new-shoot-growing stage is an important period of apple tree nutrition distribution. The objective of this study is to provide technical support for apple tree nutrition diagnosis by constructing quantitative evaluation models between the apple leaf nitrogen content during the new-shoot-growing stage and characteristic spectral parameters. The correlation coefficients between the original spectral data and the nitrogen content were calculated. Then, the sensitive bands of the nitrogen content were selected using the theory of two-dimensional (2D) correlation spectroscopy. Finally, partial least squares regression (PLSR) and support vector machine (SVM) evaluation models were established using 2 parameters: Rx (maximum spectral reflectivity in the waveband) and Sx (total spectral reflectivity in the waveband). The results showed that the sensitive bands in the 2D correlation synchronous and asynchronous spectrograms were 537–560 nm and 708–719 nm. The PLSR model can be used to estimate the nitrogen content. Compared with PLSR, SVM provided better modeling and testing results, with a larger coefficient of determination (R^2^) and a smaller root-mean-square error (RMSE). The SVM model based on Sx was a good backup method. The calibration R^2^ of the model was 0.821, its RMSE was 0.710 g·kg^-1^, the validation R^2^ was 0.768, and its RMSE was 1.019 g·kg^-1^. The SVM model based on 2D correlation spectroscopy can be used to quantitatively estimate the nitrogen content in apple leaves.

## Introduction

For apple trees, the new-shoot-growing stage is a critical period of nutrient storage and transformation. In this stage, newborn sprouts are growing rapidly and the leaves gradually mature. This stage is therefore vital for nutritional-content measurements. Nitrogen is required for plant growth [1,2]. Therefore, the ability to quickly measure the nitrogen content in the new-shoot-growing stage is important for monitoring the growth of apple trees. Compared with the chemical method, a spectroscopic technique is fast, economical, and non-destructive and provides a new method for determining the nitrogen content [3,4].

In recent years, spectral technology has been used to estimate the nitrogen content of wheat (Triticum aestivum L.) [5,6], rice (Oryza sativa L.) [7,8], and maize (Zea mays L.) [9,10]. The hyperspectral technique collects information of a target across the electromagnetic spectrum, from ultraviolet to long-infrared. The technology has the advantages of a continuous band, but the large data volume causes information redundancy [11]. Therefore, screening the sensitive waveband is one of the key techniques in hyperspectral technology [12]. It can reduce redundant features, improve the image-processing speed and improve the modeling accuracy. The sensitive wavebands have been selected using correlation analysis [13,14], principal component analysis [14], a genetic algorithm [15], sequential forward selection [16], the minimal-redundancy–maximal-relevance criterion [17], and a receiver operating characteristic curve [18]. These methods optimize the one-dimensional spectral data and screen out sensitive bands but do not analyze the source of the sensitive functional group.

Two-dimensional (2D) correlation analysis was first used in the field of nuclear magnetic resonance [19]. In 1993, Noda proposed the concept of a generalized 2D correlation spectrum, which led to its wide application [20]. The 2D correlation analysis extends the spectrum to a second dimensional space, reveals spectral feature information that is difficult to observe in the one-dimensional spectrum, and improves the spectral resolution [21]. Simultaneously, it studies different intermolecular or intramolecular interactions and confirms the origin of the functional group through analysis of the correlations among different spectral lines [19,22]. The 2D correlation analysis provides another method to screen sensitive bands. Zhang et al [23] used a 2D synchronous and asynchronous spectrogram to determine the feature waveband that represented the chlorophyll concentration of the water. They then combined a support vector machine to establish the model, where the determination coefficient of calibration (R_c_^2^) was 0.960, and the determination coefficient of validation (R_v_^2^) was 0.884. Song [24] treated water as an outer interference and applied 2D correlation spectroscopy to analyze it. Their results showed that the functional group at 1929 nm was most sensitive to water, that at 2210 nm was the second-most sensitive to water, and that at 1415 nm was the least sensitive to water.

In this study, we examined the nitrogen content of apple leaves in the new-shoot-growing stage. The objectives of this study were: i) to obtain the dynamic spectrum of nitrogen in apple leaves using the nitrogen content as the perturbation factor; ii) to select the waveband most sensitive to the changes in nitrogen content using 2D synchronous and asynchronous correlation spectroscopy; and iii) to establish an evaluation model of the nitrogen content in apple leaves using the PLSR and SVM method.

## Materials and methods

### Sampling site and sample collection

Apple leaves were collected from the Qixia City of Shandong Province, China (37°05' N to 37°32' N, 120°33' E to 121°15' E). The orchard area thereof reaches 4.3 × 10^4^ ha and the main planting variety is Red Fuji apple tree. In the second half of May 2013 (the new-shoot-growing stage), 100 Red Fuji apple trees at different growth potentials were selected randomly from 15 towns of Qixia City. Twenty leaves with different growing were collected in four orientations around the tree canopy. The study was supported by the Qixia Fruit Industry Development Bureau of China, which issued the permission for each orchard. The leaves were immediately deposited into a plastic bag, which was placed in an ice-filled foam box.

### Spectral data measurements

The reflectance spectra of apple leaves were collected using a portable object spectrometer called FieldSpec 3 (Analytical Spectral Devices Inc., Boulder, CO, USA). This portable spectrometer has a spectral range of 350 to 2500 nm. The device was calibrated with a standard whiteboard before measurements [3]. Spectral measurements were performed for the upper, middle and lower parts of each leaf. After each single leaf was fixed by the leaf clip, the spectral reflectance was measured with the built-in probe in the clip. The veins must be avoided when measuring. Ten spectra were collected for each part. The average value of all spectral values of a leaf was considered the spectral reflectance data.

### Measurement of the nitrogen content

All leaves were dried to a constant weight at 70°C in a forced-draft oven. The dried-leaf samples were ground and passed through a 0.25 mm screen. Approximately 0.2 g powder was weighed for boiling. The leaf nitrogen content was determined using the Kjeldahl method [25]. Then, 75 samples were randomly selected from 100 samples to build quantitative models; the remaining 25 samples were used to quantify the accuracy of quantitative models. The statistical results for the nitrogen content are shown in Table 1.

**Table 1:** Characteristics of the nitrogen concentration of the samples.

Samples	Observations	Maximum/g·kg^-1^	Minimum/g·kg^-1^	Mean/g·kg^-1^	Standard deviation/g·kg^-1^
Total	100	34.75	26.84	30.56	1.69
Calibration	75	34.49	26.84	30.55	1.65
Validation	25	34.75	27.04	30.56	1.82

### Two-dimensional correlation spectroscopic analysis

The spectral signal analysis was extended to two dimensions using two-dimensional (2D) correlation analysis, which can improve the spectral resolution and disassemble overlapping and mixed spectra. It can be used to extract feature information [26].

The 2D correlation analysis examined the variability of dynamic spectra. The dynamic spectrum $\tilde{y}(v,\;t)$ is defined in formula (1) [27]:

$$\tilde{y}(v,t)=\left\{ \begin{array}{l} y(v,t)-\bar{y}(v)\;T_{min}<t<T_{max}\\ 0\;else \end{array}\right.$$ (1)

where *t* is the outer interference; $T_{min}=-\frac{T}{2}$, $T_{max}=\frac{T}{2}$, T is the cycle of external interference; $\bar{y}(v)$ is the reference spectrum. In the presence of external interference, $\tilde{y}(v,\;t)$ is the original spectrum minus the reference spectrum; in the absence of an external disturbance, it is equal to zero.

The 2D correlation spectrum includes two types of synchronous and asynchronous spectra. The synchronous spectrum intensity *ø*(*v*_1_,*v*_2_) is the vector product of the dynamic spectrum intensity at different wavelengths (*v*_1_,*v*_2_). The asynchronous spectrum intensity *ψ*(*v*_1_,*v*_2_) is the Hilbert-Noda converted vector product of the dynamic spectrum intensity at different wavelengths (*v*_1_,*v*_2_) [28]:

$$ø(v_{1},v_{2})=\frac{1}{m-1}{\tilde{y}(v_{1})}^{T}\tilde{y}(v_{2})$$ (2)

$$\psi (v_{1},v_{2})=\frac{1}{m-1}{\tilde{y}(v_{1})}^{T}N\tilde{y}(v_{2})$$ (3)

where *m* is the number of spectra and *N* is the Hilbert-Noda transformation matrix.

The synchronization spectrogram represents the synergetic degree of signal strength caused by two independent optical variables with external disturbance. The correlation peak is divided into an auto-peak and a cross peak. The auto-peak is located at the diagonal of the synchronous spectrogram and is obtained from the autocorrelation of the dynamic spectral signal under the same disturbance, which represents the sensitivity of the spectral strength at this position. The cross peak at the non-diagonal represents the degree of correlation among spectra with different frequencies. If the peak at (*v*_1_,*v*_2_) is positive, the change direction of the spectral intensity is consistent; otherwise, it is different. An asynchronization spectrogram indicates the difference of the intensity variance under external disturbance. When *v*_1_ > *v*_2_, if the peaks at (*v*_1_,*v*_2_) of either the synchronous or asynchronous spectrograms are positive, *v*_1_ changes before *v*_2_; otherwise, *v*_1_ changes after *v*_2_ [18].

The nitrogen contents were considered the external interference. The 2D correlation analysis results were obtained using the Shige software, which was written by Shigeaki Morita and Yukihiro Ozaki (Shigeaki Morita, Kwansei-Gakuin University, 2004–2005). The contour line layer was set to 8. The average spectrum was considered the reference spectrum.

### Establishment and validation of the model

The PLSR and SVM methods were used to establish the hyperspectral evaluation model. As the most common modeling method, PLSR uses dimension reduction, which implies simplification of independent variables without loss of information [29,30]. This method has superior modeling effects and prediction accuracy because of its obvious advantages in processing multicollinearity and autocorrelation problems [31]. http://zh.wikipedia.org/wiki/%E7%BB%9F%E8%AE%A1%E5%88%86%E7%B1%BBhttp://zh.wikipedia.org/wiki/regression analysisSVM overcomes the problems of overfitting and trapping the minimum value via the principle of structural risk minimization. It has superior generalization ability and effectively solves the dimensionality problem [32]. The SVM method is used widely in statistical classification and regression analysis [33].

The coefficient of determination (R^2^) and RMSE were used to inspect the model. The indices were calculated from the predicted and actual values of the samples. The evaluation model with the highest R^2^ and lowest RMSE were considered the best [9].

## Results

### Spectral characteristics of different nitrogen contents

**Fig 1:**
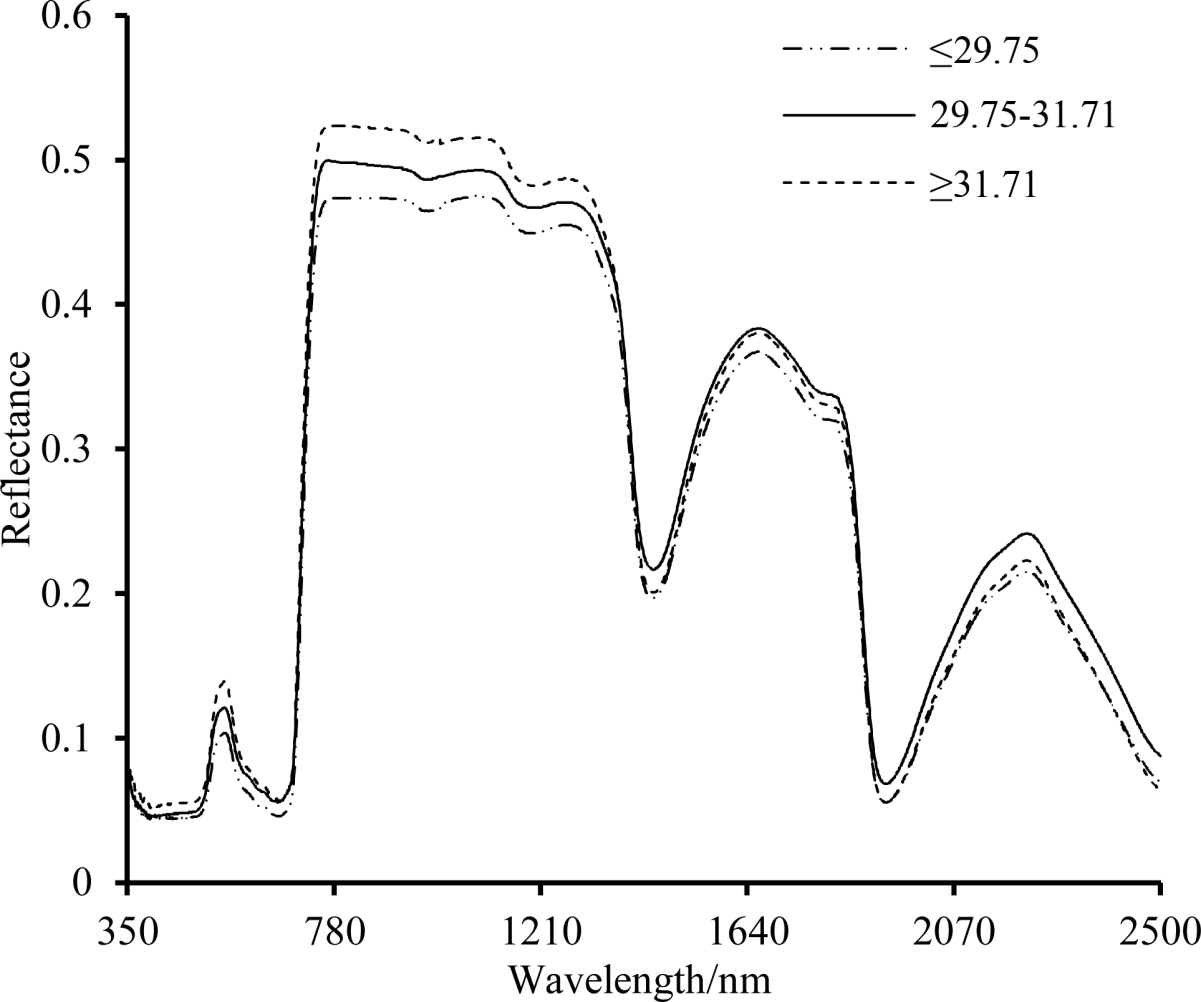
Spectral reflectance characteristics of leaves with different nitrogen concentrations.

First, 100 samples were divided into 3 groups on the basis of their average nitrogen contents: ≤ 2.975 g·kg^-1^, 2.975–3.171 g·kg^-1^, and ≥ 3.171 g·kg^-1^. The reflectance spectral curve was obtained to show the mean reflectance value in each group (Fig 1). The spectral curve shapes of all samples were similar and formed a convex parabola. The band was not considered when modeling at 350–400 nm because this region may be affected by device noise. At 430–470 nm and 620–760 nm, chlorophyll in the leaves absorbed the optical radiation for photosynthesis and formed 2 reflection troughs. The leaves with high nitrogen contents exhibited lower reflectivity than those with low nitrogen contents. In the green-light waveband, the reflection of the leaves formed an obvious reflection peak and the reflectivity value decreased with increasing nitrogen content. In the wavelength region beyond the red waveband, the reflection of the leaves sharply increased. In the 780–1300 nm range, after initially decreasing, the reflectivity increased with increasing nitrogen content. In the 1300–2500 nm region, the reflectance spectral curve formed 2 obvious absorption troughs and reflection crests, which may be related to moisture in the plant, mesophyll cell tissue and dry matter. The reflectivity showed staggering changes with increasing nitrogen content.

### Two-dimensional spectral characteristics of leaves

**Fig 2:**
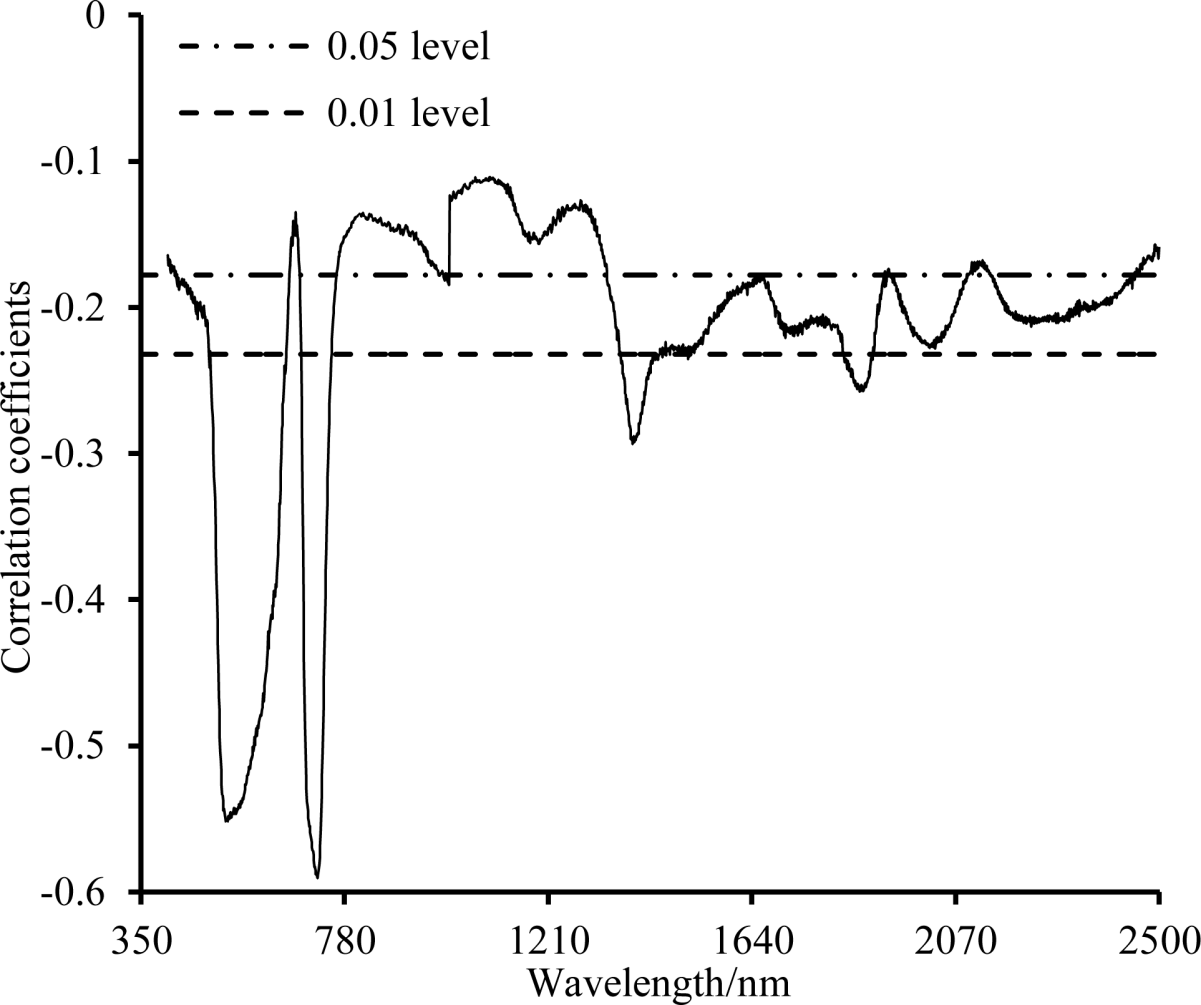
Correlation analysis between the spectral reflectance and the nitrogen concentration.

The correlation coefficient at each wavelength between the raw spectral value and the nitrogen content was calculated for the entire 350–2500 nm region (Fig 2). The nitrogen content was correlated significantly with the spectra in the 493–656 nm, 688–751 nm, 1362–1458 nm, and 1834–1895 nm regions (P </ = 0.01, r_max_ = -0.591). The 1362–1458 nm and 1834–1895 nm regions were excluded because they are strongly influenced by absorption bands associated with moisture and the atmosphere [31,34].

**Fig 3:**
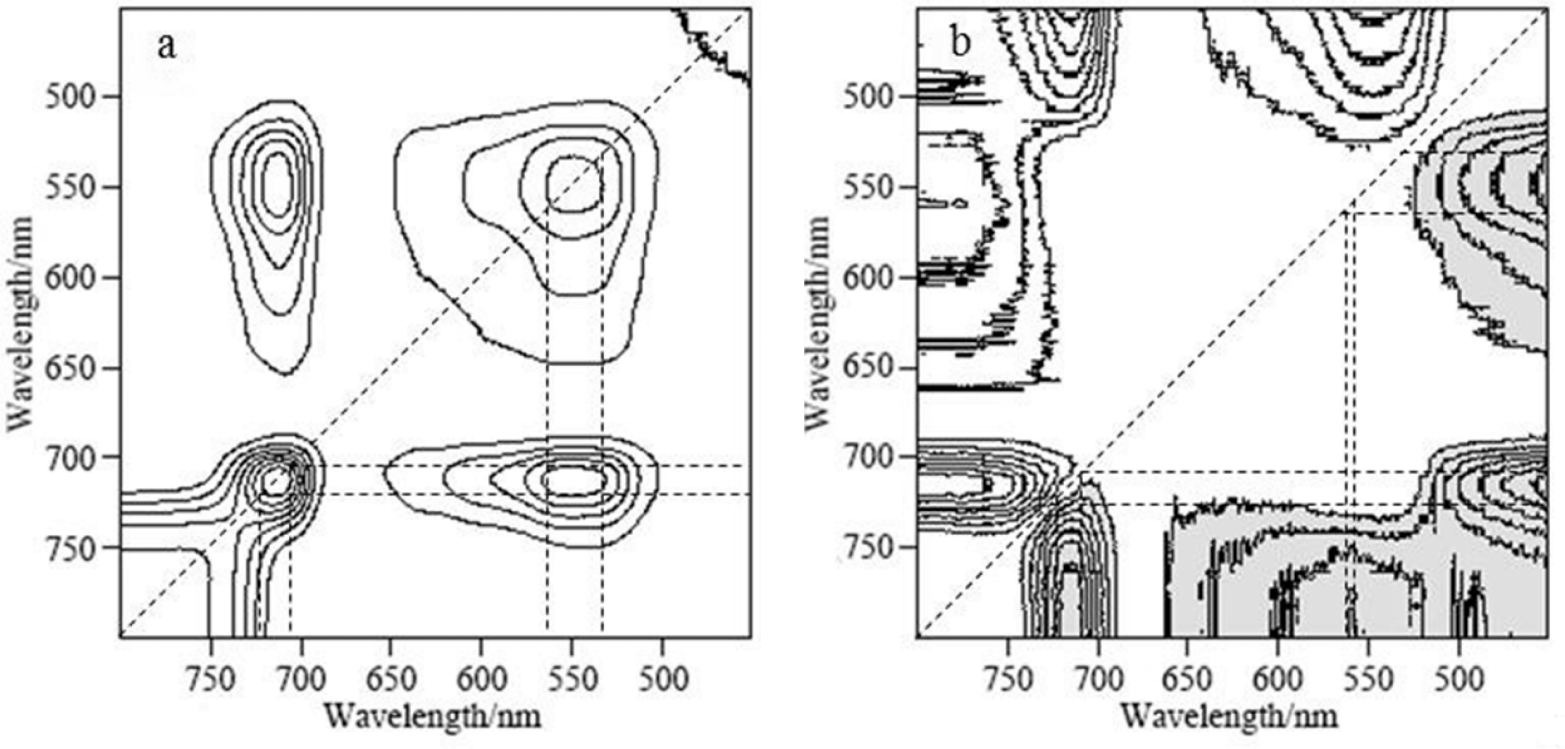
Synchronous (a) and asynchronous (b) two-dimensional correlation spectra.

Two-dimensional correlation analysis was conducted for the 493–656 nm and 688–751 nm ranges. Fig 3 shows the 2D synchronous and asynchronous spectrograms of the nitrogen content in leaves at 450–800 nm. The synchronous spectrogram contained two auto-correlation peaks at approximately 537–560 nm and 708–719 nm (Table 2); these results indicate that these two wavebands were the most sensitive to the external interference and that their spectral strengths fluctuated with the nitrogen content. Meanwhile, positive cross peaks were observed at 537–560 nm and 708–719 nm (Table 2), which indicates that the reflectivity strength corresponding to the two wavebands synchronously changed with the external disturbance and was affected by the same substance in the apple leaf. The wavebands sensitive to the chlorophyll content in apple leaves were contained in the ranges from 515 to 590 nm and from 688 to 715 nm [35]. Furthermore, Li [36] concluded that chlorophyll exhibits strong reflectivity at approximately 530–570 nm and that the reflectivity in the 700–725 nm range also is related to the chlorophyll content. Given the results of the 2D correlation analysis, the reflectivity intensity at 545–559 nm and 688–715 nm synchronously changed with the chlorophyll content. Because nitrogen is a component of chlorophyll, the nitrogen content is related closely to these two wavebands.

**Table 2:** The peak of synchronous and asynchronous two-dimensional correlation.

Peak	Horizontal axis/nm	Vertical axis/nm
Auto-correlation peak	537~560	537~560
Auto-correlation peak	708~719	708~719
positive cross peak	537~560	708~719
Cross peak	450~456	534~565
Cross peak	450~456	714~728
Cross peak	556~561	785~800
Cross peak	709~721	744~800

There are four cross peaks in the asynchronous spectrogram (Table 2). These cross peaks indicate that the functional groups that vibrated at 450–456 nm vibrated in a different direction than those associated with the bands at 534–565 nm and 714–728 nm and that the spectral strength reversibly changed under the external disturbance. The functional group associated with the band at 744–800 nm vibrated in the opposite direction than those associated with the bands at 556–561 nm and 709–721 nm. Therefore, the reflection peaks at (534–565 nm, 709–728 nm) and (450–456 nm, 744–800 nm) did not reflect the same substance in the leaf. A strong absorption trough of xanthophyll was observed at 450–456 nm [37,38]. Furthermore, Li [36] reported that the water content in apple leaves is correlated strongly with the reflection spectrum within in the 420–500 nm and 740–860 nm regions. Finally, 537–560 nm and 708–719 nm were selected as the sensitive bands for the nitrogen content estimation, consistent with previous research results [39,40]. These wavebands are the green- and red-light regions, which are typical spectral regions of plants and are consequently reliable and sensitive regions associated with the nitrogen content.

### Establishment and validation of the evaluation model

Two characteristic spectral parameters were constructed to establish the evaluation model: Rx (maximum spectral reflectivity in the waveband) and Sx (total spectral reflectivity in the waveband).

#### Partial least squares regression

The characteristic spectral parameters within the 537–560 nm and 708–719 nm regions were considered independent variables and the nitrogen content was considered a dependent variable to establish the partial least square regression model. Model (4) and model (5) are the estimation models based on the Rx and Sx characteristic spectral parameters, respectively:

$$y=5.629-7.241{Rx}_{1}-5.697{Rx}_{2}$$ (4)

$$y=5.232-0.304{Sx}_{1}-0.458{Sx}_{2}$$ (5)

For the model based on Rx, the calibration R_c_^2^, root-mean-square error of calibration (RMSE_c_), validation R_v_^2^, and root-mean-square error of validation (RMSE_v_) were 0.778, 0.773 g·kg^-1^, 0.665, and 1.378 g·kg^-1^, respectively. For the model based on Sx, the calibration R_c_^2^, RMSE_c_, validation R_v_^2^, and RMSE_v_ were 0.773, 0.782 g·kg^-1^, 0.664, and 1.368 g·kg^-1^, respectively (Table 3). The PLSR model based on the Rx parameter was slightly better than the PLSR model based on Sx. The two models can be used to estimate the nitrogen content. Although the linear model is simple and intuitive, the estimation accuracy must be improved.

**Table 3:** Establishment and validation of the evaluation models.

Modeling method	Characteristic parameter	R_c_^2^	RMSE_c_/g·kg^-1^	R_v_^2^	RMSE_v_/g·kg^-1^
PLSR	Rx Sx	0.778 0.773	0.773 0.782	0.665 0.664	1.378 1.368
SVM	Rx Sx	0.819 0.821	0.703 0.710	0.759 0.768	1.102 1.019

R_c_^2^: determination coefficient of calibration; R_v_^2^: determination coefficient of validation; RMSE_c_: root-mean-square error of calibration; RMSE_v_: root-mean-square error of validation

#### Support vector machine

A multivariate nonlinear model was established to take the characteristic spectral parameter corresponding to the 537–560 nm and 708–719 nm ranges as the condition attributes and the nitrogen content as the decision attributes of the SVM model. Through parameter optimization and model verification, the SVM type and the kernel function type were determined to be v-SVR and RBF, respectively. Other model parameters are shown in Table 4.

**Table 4:** Support vector machine regression model parameters.

Degree	Gamma	Coef0	Nu	Epsilon	Cashesize	Cost	Shrinking	Prob	P
3	0.5	0.001	0.5	0.001	100	1	1	1	0.01

Degree: set degree in kernel function; Gamma: set gamma in kernel function; Coef0: set coef0 in kernel function; Nu: set the parameter nu of nu-SVC, one-class SVM, and nu-SVR; Epsilon: set tolerance of termination criterion; Cashesize: set cache memory size in MB; Cost: set the parameter C of C-SVC, epsilon-SVR, and nu-SVR; Shrinking: whether to use the shrinking heuristics, 0 or 1; Prob: whether to train a SVR model for probability estimates, 0 or 1; P: set the epsilon in loss function of epsilon-SVR.

**Fig 4:**
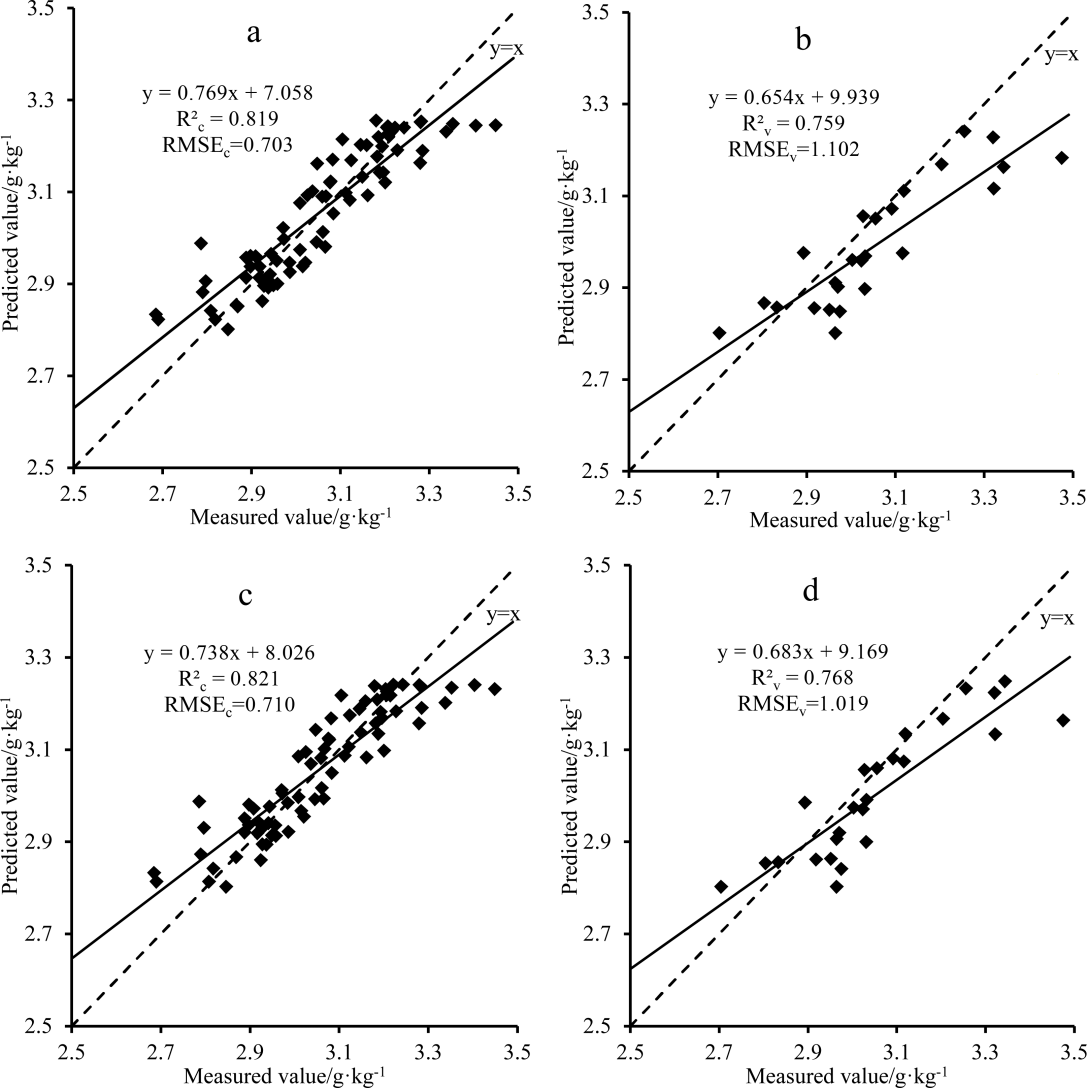
Comparison of the SVM measured values and the values predicted on the basis of the (a) calibration with Rx, (b) validation with Rx, (c) calibration with Sx, and (d) validation with Sx.

For the model based on Rx, the calibration R_c_^2^, RMSE_c_, validation R_v_^2^, and RMSE_v_ were 0.819, 0.703 g·kg^-1^, 0.759, and 1.102 g·kg^-1^, respectively. For the model based on Sx, the calibration R_c_^2^, RMSE_c_, validation R_v_^2^, and RMSE_v_ were 0.821, 0.710 g·kg^-1^, 0.768, and 1.019 g·kg^-1^, respectively (Table 3). The SVM model based on the Sx parameters was slightly better than that based on Rx. The R^2^ of the SVM models were larger than the R^2^ of the PLSR models, and the RMSE of the SVM models were smaller the RMSE of the PLSR models. The SVM models were better than the PLSR models. According to the established model, we constructed a 1:1 relationship diagram between the measured and predicted values to show the reliability and consistency of the SVM model; the result is shown in Fig 4. The measured and predicted values of the modeling and testing were approximately 1:1. The SVM model can be used to estimate quantitatively the nitrogen content and represents an effective 2D correlation spectrum technology for the evaluation of the nitrogen content in leaves.

## Conclusions

The nitrogen content was considered an external interference to obtain the dynamic spectrum of nitrogen in leaves. The nitrogen-sensitive bands were those at 537–560 nm and 708–719 nm in the synchronous or asynchronous spectrograms. The SVM models were better than the PLSR models, with larger R^2^ and smaller RMSE values than PLSR models'. The SVM model with the 2D correlation analysis and Sx served as the optimal method to estimate the nitrogen content in apple leaves in the shoot-growing stage (R_c_^2^ = 0.821, RMSE_c_ = 0.710 g·kg^-1^, R_v_^2^ = 0.768, RMSE_v_ = 1.019 g·kg^-1^). The model achieved a notably high accuracy and provides technical support for the scientific management of the nitrogen content. The leaf nitrogen and spectral data must be measured in the estimation process, which inevitably may involve human error. In future work, we will establish the leaf optical model to test the effect of the estimation model, thereby providing a theoretical basis for the development of spectral analysis technology for apple leaves.

## Supporting information

S1 Data Spectra and nitrogen content of apple leaves. (XLS)
